# Spirituality, religiosity, and their representations for people living with HIV: daily life and its experiences^*^

**DOI:** 10.1590/1980-220X-REEUSP-2022-0394en

**Published:** 2023-05-26

**Authors:** Virginia Paiva Figueiredo Nogueira, Antonio Marcos Tosoli Gomes, Magno Conceição das Mercês, Pablo Luiz Santos Couto, Sérgio Donha Yarid, Priscila Cristina da Silva Thiengo de Andrade

**Affiliations:** 1 Universidade do Estado do Rio de Janeiro, Faculdade de Enfermagem, Rio de Janeiro, RJ, Brazil.; 2 Universidade do Estado da Bahia, Departamento de Ciências da vida, Salvador, BA, Brazil.; 3 Universidade Estadual do Sudoeste da Bahia, Programa de Pós-Graduação em Enfermagem e saúde, Jequié, BA, Brazil.

**Keywords:** Spirituality, Religion, HIV, Acquired Immunodeficiency Syndrome, Nursing, Espiritualidad, Religión, VIH, Síndrome de Inmunodeficiencia Adquirida, Enfermería, Espiritualidade, Religião, HIV, Síndrome da Imunodeficiência Adquirida, Enfermagem

## Abstract

**Objective:**

To describe the process of living with HIV/AIDS in the daily life of people living with HIV in its interface with the social representations of spirituality and religiosity.

**Method:**

Qualitative research, supported by the theory of social representations. A semi-structured interview was carried out with 32 people undergoing treatment for HIV in an outpatient clinic specialized in HIV/AIDS. Analysis carried out with the support of *software* IRAMUTEQ.

**Results:**

Participants were mostly men, aged over 51 years, Catholic, and living with the virus for more than 10 years. IRAMUTEQ generated three classes, in which the influence of spirituality and religiosity as a promoter of strength to face the infection and the difficulties in the process of coping with the diagnosis was observed, as well as the importance of the support network, and the naturalization of HIV/AIDS.

**Conclusion:**

The participants make associations between spirituality and the transcendent and divine; religiosity was anchored to religion and its experience, with both being a source of support and strength. Therefore, it is important to make room for the patient to talk about their spiritual/religious needs.

## RESUMO

**Objetivo:**

Descrever o processo de viver com HIV/Aids no cotidiano de pessoas vivendo com HIV em sua interface com as representações sociais da espiritualidade e da religiosidade.

**Método:**

Pesquisa qualitativa, apoiada pela teoria das representações sociais. Realizou-se entrevista semiestruturada com 32 pessoas em tratamento para HIV num ambulatório especializado em HIV/Aids. Análise realizada com suporte do *software* IRAMUTEQ.

**Resultados:**

Os participantes foram em sua maioria homens, com idade superior a 51 anos, católicos e vivendo com o vírus há mais de 10 anos. O IRAMUTEQ gerou três classes. Nestas, viu-se a influência da espiritualidade e da religiosidade como promotora de força para o enfrentamento da infecção, assim como das dificuldades no processo de enfrentamento do diagnóstico, e a importância da rede de apoio e a naturalização do HIV/Aids.

**Conclusão:**

Os participantes fazem associações da espiritualidade ao transcendente e ao divino. A religiosidade foi ancorada à religião e à sua vivência, ambas sendo fonte de apoio e força. Por isso, é importante abrir espaço para o paciente falar sobre sua necessidade espiritual/religiosa.

## RESUMEN

**Objetivo:**

Describir el proceso de vivir con SIDA en el cotidiano de personas viviendo con VIH en su interfaz con las representaciones sociales de la espiritualidad y de la religiosidad.

**Método:**

investigación cualitativa, apoyada por la teoría de las representaciones sociales. Se realizó entrevista semiestructurada con 32 personas en tratamiento para VIH en un ambulatorio especializado en VIH/SIDA. Análisis realizado con soporte del software IRAMUTEQ.

**Resultados:**

Los participantes fueron en su mayoría hombres, con edad superior a 51 años, católicos y viviendo con el virus hace más de 10 años. El IRAMUTEQ generó tres clases, en las que se notó la influencia de la espiritualidad y de la religiosidad como promotora de fuerza para el enfrentamiento de la infección. Sino también, las dificultades en el proceso de enfrentamiento del diagnóstico, la importancia de la red de apoyo y la naturalización del VIH/SIDA.

**Conclusión:**

Los participantes hacen asociaciones de la espiritualidad al transcendente y al divino, la religiosidad fue ancorada a la religión y a su vivencia, ambas siendo fuente de apoyo y fuerza. Por eso, es importante abrir espacio al paciente hablar sobre su necesidad espiritual/religiosa.

## INTRODUCTION

The human immunodeficiency virus (HIV) infection can still be considered a serious public health problem, both for the difficult prevention and for all the psychosocial aspects surrounding it. In Brazil, in 2020, 32,701 new cases of infections were diagnosed and in the period from 1980 to June 2021, 1,045,355 cases of AIDS were detected in the country ^(1)^.

Therefore, it is important to investigate ways to help people living with HIV (PLHIV) in coping with the diagnosis, the treatment and daily living with the change in lifestyle, and in facing fear of the disease manifesting itself, fear of discovering the diagnosis and suffering prejudice^(2–4)^.

In recent years, research has been conducted on spirituality and religiosity in the context of health and illness, including living with HIV/AIDS. Some studies show how this dimension can promote strength, help in accepting and coping with living with the infection and with the possibility of the syndrome appearing, as well as establishing a beneficial relationship between quality of life and spiritual well-being^(5,6)^.

Another important aspect when studying the subject is the adherence to antiretroviral therapy, a psychosocial dimension that can be a predictor of adherence to antiretroviral drugs. There is still no consensus on the meaning of this influence, but investigation and comprehension of the phenomenon of spirituality and religiosity for this social group^(7)^ is recommended. It should be noted that there are differences between the concepts of spirituality and religiosity. Although there is no single definition of spirituality, it can be considered as a personal quest to reach contact with the divine, the transcendent, as well as understanding the meaning of life, a dimension that may or may not be related to religious practices. Religiosity can be understood as adherence to religious beliefs and practices, that is, how much one believes and practices a religion. It has an intrinsic dimension when there are personal religious practices such as prayers, reading of religious texts, and an extrinsic dimension when it is lived together with the religious community and its rites^(8)^.

Dealing with religiosity and spirituality in the context of HIV can be a delicate task, since religious discourses on behavior of vulnerability to HIV can have a moral nature and can be a challenge in communication about sex education in religious communities, serving as a barrier that hinders prevention and coping in case of a positive result for the infection. At the same time, the religious community can, through its members, offer support, help in coping, which generates well-being and serves as a social support network, facilitating the treatment and management of the infection^(9)^.

This way, spirituality and religiosity are objects circulating within the PLHIV group. They are present in conversations, images and social thinking. In the context of living with HIV/AIDS, it is important to know these social representations (SR) and how they can influence people’s lives. Based on the above, this article aims to describe the process of living with HIV/AIDS in the daily lives of people living with HIV in its interface with the social representations of spirituality and religiosity.

## METHOD

### Theoretical Framework

The theory of social representations, elaborated by Serge Moscovici, developed by him and also worked by other researchers such as Denise Jodelet, Jean-Claude Abric, in France, Celso Pereira de Sá, in Brazil, among others, will be the basis of this study. It was defined by Moscovici as a psychosociology of knowledge, a particular type of knowledge that has the attribute of reproducing, at cognitive level, the properties of an object, of fusion between the concept and the perception of the concrete and imagetic character, as well as the significant value of qualities that are intrinsic and extrinsic to the object. The representation can be seen as a reflection of the object and the subject’s activity. In its range it is social^(10)^ and is widely used to understand objects related to the field of health.

The representations are social because the world is shared among the different people who compose it, serving as support for each other, in convergence or coexisting in a conflicting way, but who seek to understand, manage and face the world. Thus, when representing, man seeks to apprehend the reality surrounding him, either through communication, gestures, or behaviors of the world surrounding him^(11)^.

### Design of Study

Descriptive and exploratory study with a qualitative approach^(12)^ and the theoretical and methodological support of the Theory of Social Representations in its procedural approach. The criteria of the international protocol *Consolidated Criteria for Reporting Qualitative Research* (COREQ) for qualitative research were considered.

### Population and Study Local

Data collection was carried out in a specialized outpatient service (SAE) in HIV/AIDS of a state university hospital in the city of Rio de Janeiro. Thirty-two people living with HIV, attended at the unit, participated. They were introduced to the research and invited to participate while waiting for their consultation or after it. People who agreed to participate were sent to a room to be interviewed in privacy. Patients attended at the service who were over 18 years old were included and those who were not in good health conditions to answer the questions were excluded. Participants were between 20 and 65 years old and had been receiving treatment at the unit for at least one year.

The number of 32 participants who responded to the interview was reached based on the methodological recommendations by Oliveira, Marques, Gomes, and Teixeira^(13)^ when considering that, in social representation studies, this number of interviews can be used if they are carried out in depth. The tutorial for using IRAMUTEQ^(14)^ also advises that 20 to 30 interviews are sufficient in cases of longer texts, because when the group is homogeneous, sample saturation can be reached.

### Instruments Used to Collect Information and Data Collection

The script used to conduct in-depth interviews was the structured interview^(15)^ which occurs as a conversation about the object of study in which the open questions allow the interviewee to discuss the subject, exploring issues contained therein, with the possibility of emergence of unpredictable questions through its course. The script also contained questions for the sociodemographic characterization of the participants, as well as questions about spirituality, religiosity and living with HIV/AIDS.

The interviews were conducted by the first author, a doctoral student during the data collection period, and by two master’s students. The three researchers were instructed on how to conduct the interviews by the advisor. Interviewers and participants did not know each other previously. The first contact took place at the time of the approach, at the outpatient clinic, to invite them to participate in the research. There were no withdrawals after acceptance to participate in the interview and few refused to participate. About five people reported being worried about missing their consultation if they participated in the research or being in a hurry to leave after the consultation. The interviews were audiorecorded, started after the participants signed the informed consent form, and lasted an average of 30 minutes.

This work comes from a study that had a long period of data collection due to its design, extending from March 2015, with the beginning of the doctorate of the first author, until October 2017, and the interviews were collected from January to March 2016.

### Data Analysis and Treatment

The data obtained for the sociodemographic characterization were organized in *Microsoft Excel*® spreadsheets and were later analyzed using the software IBM® SPSS® by descriptive statistical analysis through simple frequencies.

The interviews were fully transcribed, saved in an *Open Office*® single file. Then, lexical analysis was performed with the support of *software* IRAMUTEQ (*Interface de R pour les Analyzes Multidimensionnelles de Textes et de Questionnaires*) version 0.7 alpha 2 which is free and has an open source. It is anchored in R software and the Python language. When submitting the corpus to analysis by IRAMUTEQ, the lexicographical analysis takes place, which indicates and reformats the text units so that they are identified in terms of number of words, average frequency, and *hapax* (words with frequency one), searches the vocabulary, and reduces words based on their roots (reduced forms), creates the dictionary of reduced forms, and identifies active and supplementary forms^(14)^.

For this study, descending hierarchical classification (DHC) was used, in which the text segments are classified according to their respective vocabularies and their set is divided according to the frequency of the reduced forms. By crossing segments of texts and words, the definitive classification is obtained, the *software* organizes the data analysis into a dendogram that illustrates the relationships between classes^(14)^. The idea of a relationship between linguistic context and collective representation or between context unit and typical context is at the base of the software’s operation. Elementary context unit (ECU) can be understood as a type of elementary representation, that is, a meaning or a minimal statement of a speech that can be understood as a thought of a psychic individual, which refers to an object and the subject itself, at the same time, allowing the formation of the representation of an object. For each class, a list of significant words for the class is generated based on the chi-square test^(13)^. It should be noted that this type of analysis, to be useful for the classification of any textual material, requires the classification or use of at least 75% of the text segments^(14)^.

### Ethical Aspects

Participation in the research was voluntary based on the free and spontaneous acceptance of its objectives, followed by the signing of the Free and Informed Consent Form. The project was approved by the Research Ethics Committee (CEP) of the State University of Rio de Janeiro under opinion no. 699.220, in 2014. As this work is part of a larger study and had an extensive period of data collection, we also included CEP opinion no. 2.660.127, approved in 2018. The research respected the ethical-legal procedures that constituted the fulfillment and use of the ethical values established by Resolution 466/2012 of the Ministry of Health^(16)^. In addition, the participants’ testimonies will be coded by the interview number, sex, religion and class chi-square score. (id_00, sex, religion, score: chi-square).

### Data Availability

Supplementary research material, as recommended by Open Science, can be found in the repository Scielo data: https://doi.org/10.48331/scielodata.HNQUA3.

## RESULTS

### The Participants’ Characterization

Regarding the participants’ characterization, it should be noted that most were men (68.7%), aged over 51 years (37.5%). Regarding religion, most declared themselves Catholic (31.25%), 34.3% had been diagnosed for up to 10 years. On the other hand, 31.3% had lived with HIV between 17 and 25 years. The result of the characterization of the participants is shown in Table 1.

**Table 1:** Sociodemographic characteristics of people living with HIV treated at a specialized outpatient service (n = 32). Rio de Janeiro, RJ, Brazil.

Variables	n	(%)
**Sex**		
Male	22	68.7
Female	10	31.3
**Age**		
21 to 41 years	9	28.1
42 to 50 years	10	31.3
Over 51	12	37.5
Not informed	1	3.1
**Religion**		
Catholic	10	31.25
Evangelical	8	25
Without religion	7	22
Spiritist (Kardecist)	6	18.75
*Candomblé*	1	3
**Diagnostic time**		
Up to 10 years	11	34.3
11 to 16 years	10	31.3
17 to 25 years	10	31.3
Not informed	1	3.1
**Use of antiretrovirals**		
Yes	31	96.9
No	1	3.1
**Have you ever abandoned treatment?**		
Yes	11	34.4
No	21	65.5
Total	32	100

*Procedural analysis of the social representations of spirituality and religiosity for people living with HIV/AIDS.*

The lexical content analysis performed in the Reinert method command of software IRAMUTEQ, as described in the method, presented a corpus of analysis consisting of 32 starred lines corresponding to the interviews that were processed and analyzed by the software, obtaining a use of 87.67% of the *corpus*.

**Figure 1:**
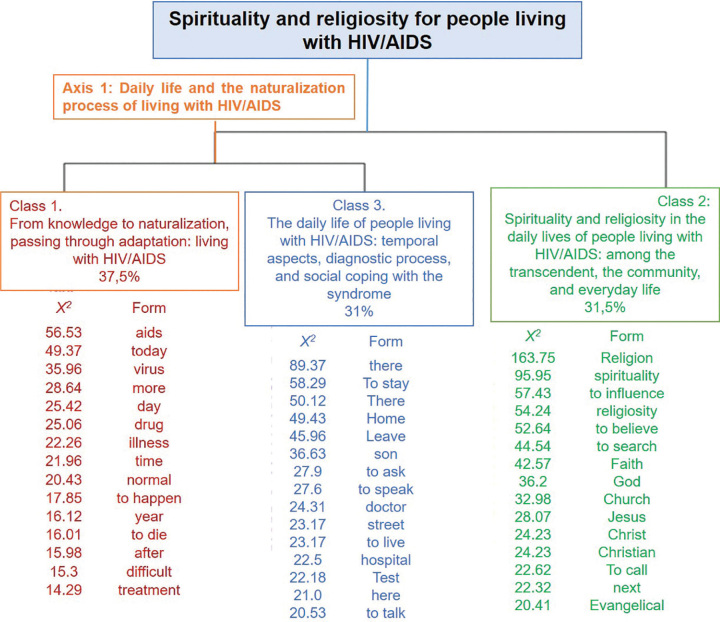
Schematic presentation of the axes and classes named by the characteristic words of the class and chi-square value of the social representation of spirituality and religiosity for people with HIV (n = 32). Rio de Janeiro, RJ, Brazil, 2020.

The *corpus* generated three classes, as shown in the dendrogram in Figure 1, below, which displays the DHC and illustrates the relationships established between the classes, which can be read from right to left, that is, first, the corpus was divided into two subgroups, forming, at this first moment, class 2. Subsequently, the material underwent another division, generating classes 1 and 3. The details of the classes generated by the analysis of the content of the Social representations of spirituality and religiosity for people living with HIV/AIDS was carried out in the order in which the divisions between classes occurred and will have the results shown below.

#### Class 2 – Spirituality and Religiosity in the Daily Lives of People Living with HIV/AIDS: Among the Transcendent, the Community, and Everyday Life

I n this class, the elementary context units presented themes such as the meaning of religiosity, religion and spirituality. The general content of the class brought the thoughts of the interviewees about religiosity and spirituality, cross-cutting issues in their lives, mainly in relation to coping with living with HIV and the struggle not to develop AIDS.

Spirituality and religiosity emerged in the ECU, often as synonyms. Religiosity was associated with religion, and is characterized by promoting a connection with God*. I think religiosity has to do with someone who genuinely wants to reconnect with the divine. This one I bet it is, this is what I’m looking for, religiosity with spirituality, not religion.* (id_135, male, no religion; score: 375.95).

For the interviewees, the concepts of spirituality and religiosity can merge at times, but in common sense there is the idea that religiosity is related to a religion and its dogmas, rituals. Spirituality, on the other hand, is related to the search for contact with a transcendent being despite religion, as expressed in the following excerpt. *Spirituality, I think it is, I changed the answers, religiosity would be something more connected to religion and spirituality connected to a superior being independent from that religion*. (id_144, male, no religion; score: 363.71).

Spirituality can influence the lives of PLHIV by allowing a better acceptance of the infection and, from there, there is an opening to better understand what HIV is. Religiosity can also have a positive influence on life, as the following excerpts illustrate. *Spirituality influences my life a lot, a lot, because I believe that when I sought spirituality I managed to have more understanding of what it is and I was able to accept it better and when I started in religion.* (id_131, female, spiritist; score: 425.97).

*Religiosity is having faith in the sacred that exists within you. Religion, if I say that it influences my life with AIDS, I’ll be lying. I think it influences my life.* (id_146, female, *Candomblé*; score: 351.66).

The ECU also brought in their content faith and belief in healing. And despite this hope, respondents are clear about the importance of adherence to treatment to stay healthy. *How does religion influence my life? Well, I’m evangelical, so in my case, I get attached to prayers. I don’t stop taking my medicine because healing is from God*. (id_143, male, evangelical; score: 308.73).

Spirituality was highlighted for its influence on the interviewees’ lives, for the connection with God and for the promotion of feelings that generate strength and help in facing difficulties. The experience of religion can promote an effect of strength in the person who believes, influencing their thoughts and the way they act, to help in facing adversities, as the excerpt illustrates: *Because I seek my answers within myself. Religion teaches that, to be closer to God, to have strength. You don’t have the strength, it gives you that, strength*. (id_138, male, spiritist; score: 286.18).

### Axis 1: Living with HIV/AIDS: Discovery, Acceptance, Treatment, and Everyday Life

#### Class 1 – the Daily Life of People Living with HIV/AIDS: Temporal Aspects, Diagnostic Process, and Social Coping with the Syndrome

The class is characterized by the dominance of the ECU on the daily life experienced by the interviewees with their families and the professionals who assist them in the treatment, on the process of knowing the diagnosis and on communicating it to the family.

The ECU of the interviewees addressed the process of diagnostic discovery, illustrating the difficulty of having counseling in the 1990s and how there are still cases of revealing the diagnosis without counseling, a fact that makes the process of accepting the infection more difficult. The following excerpts illustrate this moment: the first statement narrates what happened in 1996 and the second in 2015. *There was no advice, no. I went without knowing it, when I took that test there, I’m not a fool, and when I saw that there I was terrified*. (id_125, male, evangelical; score: 206.76). Another participant said: *I left the house in a daze and I needed someone to help me, to give me a kind word and I was so dazed on the street of the federal university. I left there and I had no strength.* (id_151, male, no religion; score: 231.19).

Another aspect that appeared in the context units was the coping related to the different stages of acceptance of the diagnosis, with the thought of imminent death, blaming the other for contamination, and mobilization of affections expressing violent thoughts, as in the excerpt: *I immediately thought I would die. Then not, I got depressed, and the feeling was: you see a woman on the street, you know, you want to break her neck, because it was with a woman, I had no other relationship except with a woman.* (id_125, male, evangelical; score: 242.73).

A contemporary issue that has to be faced is that many babies who were infected through vertical transmission by their mothers are currently teenagers or young adults. Thus, these young people had to face living with HIV since birth and, at some point in their lives, had to reveal their diagnosis, especially in stable relationships with serodiscordant partners. *She asked me at one point, like, I had already been dating her for almost two years, I went and told her. She was, wow, surprised, but I’m still with her today, it’s been almost three years now*. (id_139, male, Catholic; score: 229.81).

Another evidenced result was that living with HIV and coping with it in everyday life generate some disorders. From the moment you receive the news of HIV infection, you have to make regular visits to health services, for consultations and routine exams, to get medication, or even consultations to make adjustments in the drug doses due to side effects of antiretrovirals, among other situations. This picture can generate anxiety before the consultation, as exemplified in the excerpt: *Pressure goes up whether you want it or not, with medication or without medication, it goes up, then you get there, check the pressure, you’ll stay there waiting for the pressure to go down, you can’t leave like that*. (id_126, female, spiritist; score: 267.31).

PLHIV may also need to conceal the infection as a social survival strategy. Frequently, when disclosing their HIV-positive diagnosis, they have to face stigma and prejudice. Thus, the social support network is fundamental for emotional support and to help in everyday life. They often have friends and health professionals in their support network.

*The thing is the prejudice that exists, but it exists in all places, it exists everywhere, not just in my religion. I have a friend who is a Christian, wow, he was expelled from the church ... he suffered a lot. We had to talk to him a lot, ... he carried his faith in a way, with so much affection, with all due respect, he was expelled just because he found out he had HIV, but we are taking it in a good way, we talk to him, we are talking and fighting with him to be able to... Because he panicked after all that, he was out of work, he was kicked out of religion, for him his world collapsed, all at once. Then we talk to him, help him, one gives him food stamps in a month, another one takes him to the doctor, we go there and we fix the house for him, another goes there to accompany him on a daily basis for him to take the medicine.* (id_140, male, spiritist; score: 258.29).

#### Class 1 – from Knowledge to Naturalization, Passing Through Adaptation: Living with HIV/AIDS

This class has context units reflecting the social representation of AIDS for the group, how they think about HIV/AIDS, treatment, death and other related issues. For the group, AIDS is considered both the immunodeficiency syndrome, with death caused by opportunistic infections, and a chronic disease, whether compared to chronic degenerative diseases or emerging and neglected diseases, such as dengue, Zika, and leprosy.

*So today there is a disease that no longer kills. You don’t die of AIDS, you die of everything but AIDS. It’s just opportunistic, it takes advantage, since it’s bad, so let’s go, let’s make the situation worse.* (id_126, female, spiritist; score: 201.59).

*So that’s what AIDS is for me, it’s a disease like any other. Even today, treatable, chronic, not cool. There is a number of other issues that entail the use of antiretrovirals.* (id_135, male, no religion; score: 175.52).

*Aids is a disease, an infectious disease like dengue fever, the zika virus, like leprosy in the Middle Ages. I don’t remember exactly what it was called, but today people commonly call it leprosy.* (id_129, female, no religion; score: 145.36).

The results also show that even considering living with HIV as a chronic condition, people should take precautions, as antiretroviral treatment has its issues, such as adaptation to it and side effects. *At the beginning it is difficult, but not today, today I don’t have any more difficulties because I got used to it. I already take it as a normal thing, I don’t have that concern anymore. I lead a normal life, concerning time, it’s twelve years.* (id_133, male, Catholic; score: 174.22).

Respondents consider that, currently, people see the person living with HIV/AIDS more naturally, as in previous years they kept themselves distant due to negative thoughts about the virus and the syndrome, as well as about people living with HIV.

*I think they see it as a normal thing nowadays. Before, about ten, fifteen years ago, people looked at a person with the AIDS virus, they measured the distance. Currently it is different, they hug you, kiss you.* (id_132, male, evangelical; score: 229.34).

## DISCUSSION

The results revealed the interfaces of the process of living with HIV and the social representations of spirituality and religiosity in everyday life. With regard to religiosity and spirituality, it should be noted that, from the interviews, in the context of common sense, their concepts intertwine and are often used as synonyms, a result also found in a study with elderly people living with HIV^(17)^. Unlike what happens in the context of scientific knowledge that seeks deeper definitions of objects, common sense knowledge seeks to define them through experience and practical feelings.

When looking for definitions for religiosity, a study^(18)^ cites classic authors who write about religion, such as sociologists Émile Durkheim, Max Weber, and contemporary authors such as Harold Koenig. From these, it is observed that religion has different meanings and dimensions, it is an organized system of beliefs with doctrines, rituals and symbols, it is practiced by the group that has the same belief, but shares, as mentioned, different dimensions, from the most personal practices to the community ones. Religiosity follows this individual path through religion to the encounter with the transcendent in which one believes. Religiosity can be interpreted as the multiplicity of ways of accessing, expressing, and embodying the dimension of the person’s spiritual beliefs that permeates religious, personal and institutional practice^(19)^.

Regarding spirituality, it can be considered that there is no single definition. Many authors have written on the subject proposing different definitions, some relate spirituality to a dynamic and intrinsic aspect of humanity that seeks meaning and contact with the transcendent^(18)^, others define spirituality as the pursuit of an experience with the divine through connection with others, with the creation or achievement of something and with the sacred transcendent, either through a religious or non-religious expression^(20)^.

Spirituality can exert a positive influence on the lives of those living with HIV, allowing better understanding of life in the process of living with the infection. Simultaneously, spirituality can provide strength to face the difficulties of the treatment and be a bridge between the human being and the transcendent (God, divinity, *orixás*, for example), regardless of religious practice. In this study, God was a strong element in the representation of spirituality and religiosity, a being that supports and is part of the interviewees’ lives. The theistic group can be considered, either because they profess a Christian religion or because belief in God is very strong in our culture, facts that influence social representations. Thus, faith in God and prayers can also be a strategy to face living with HIV infection^(17, 21)^.

A study^(22)^ investigating works on spirituality and religiosity and health markers found that there are positive associations between different religious practices and the CD4 T cell count, as well as a decrease in the progression of HIV infection. In addition, patients who have a good social support network and those without depressive symptoms showed better adherence to treatment. Therefore, it was also observed that aspects of intrinsic and extrinsic religiosity from practices such as prayers and participation in religious celebrations could help in reducing the progression of the infection by the HIV and the depressive symptoms.

As shown in the results, there was a positive relationship between the group’s thinking and its practices in relation to spirituality and religiosity and coping with living with HIV. There is even a belief, based on religion, that God can heal, but with professional guidance there is also clarification that one cannot stop taking medications for the success of the treatment and to improve the quality of life. These findings corroborate studies that had as a result that religiosity and spirituality strengthen people with HIV in the face of their condition of living with the infection, helping them to renew their hope and contributing to a life with greater well-being^(17, 23)^.

Regarding the social representations of AIDS, studies show that these representations have been changing, which corroborates the findings of this work. For the participants, living with HIV is currently having a chronic disease compared to hypertension and diabetes due to the need for chronic use of medication and constant health monitoring. However, elements such as fear and prejudice are still very present in the daily lives of people living with HIV: fear of revealing the diagnostic condition, fear of having opportunistic diseases, fear of pregnant women of passing the virus on to their child, and fear of prejudice due to the stigma of AIDS^(3, 17, 24, 25)^.

Finally, another issue that emerged was about disclosing the diagnosis to the partner. The findings of this research corroborate another study carried out with women of different generations living with HIV, in which the younger ones agreed to reveal their condition to their partner and family, which did not occur with women of other ages^(26)^. This way of coping with the infection may be anchored in the representations of AIDS, as people who have seen well-known singers and actors become ill as a result of the syndrome possibly have a representation of AIDS permeated by elements such as death, prejudice, and fear of stigma. And younger people born after the establishment of antiretroviral therapy possibly see AIDS and HIV infection as a chronic illness, manageable with medication.

### Study Limitations and Implications for Scientific Knowledge in the Area of Health and Nursing

A limitation of this study can be the fact that it was carried out in only one health unit in the city of Rio de Janeiro, a very important reference unit for the State. However, as this work is derived from a larger study, the data collection of the general material had three stages, the second being the interview. This way, data collection became long and short breaks were taken between one collection and another so that the patients in the scenario would not get tired of our presence in the unit and of the invitation to participate in the research.

Knowing the thoughts of people who receive nursing care about spirituality and religiosity is related to the context of holistic care. When one considers that the biopsychosocial and spiritual aspects are present in the life stages of individuals, including illness, it can be inferred that this study can have good implications for health and nursing care.

When investigating these representations, it was observed that religion is also related to culture, to the context experienced by participants in society. For a few, religion has a negative connotation, possibly due to the dramas experienced, the first representations of AIDS, but for the group there is a broader meaning that concerns the positive impacts of the practice of religiosity and spirituality, which is the search for contact with God or another superior being.

Thus, opening space to listen to the patient about this aspect of his/her life, when necessary, can lead to a greater bond between the professional and the client, as well as greater understanding and openness to talk about intimate aspects that may interfere with the success of the treatment, quality of life, and their physical and mental health.

It is observed that interest in the subject has been increasing in recent years due to the growing number of studies on the subject and events in the area. Therefore, it is important that training and professional updating courses can include the subject to equip professionals to work, in the best way, this dimension of care so little addressed in practice.

## CONCLUSION

The process of experiencing HIV/AIDS in the daily life of the interviewed group and the interface with the social representations of spirituality and religiosity revealed that the participants make associations between spirituality and the transcendent and the divine, and religiosity was anchored to religion and its experience, with both being a source of support, acceptance and strength.

Through the ECU, it could be seen that the religious/spiritual dimension and its practices, either intrinsic or extrinsic, are capable of providing positive aspects in the participants’ physical and mental health.

Discovering the diagnosis and coping with living with HIV brings with it a set of attitudes, practices and feelings. Therefore, encouraging the patient to talk about his/her feelings and asking about his spiritual/religious need can help the professional to understand the patient’s thinking about the two objects and the relationship with his/her illness. For this, it is possible to leave an open space to hear about this dimension, either during history taking or the nursing consultation. This way, one can talk about the meaning of HIV, spread knowledge about the infection and treatment, and help the patient in this cooping.
